# What Happens to Underprepared First-Time-in-College Students When Developmental Education is Optional? The Case of Developmental Math and Intermediate Algebra in the First Semester

**DOI:** 10.1080/00221546.2017.1390970

**Published:** 2017-11-21

**Authors:** Toby Park, Chenoa S. Woods, Shouping Hu, Tamara Bertrand Jones, David Tandberg

**Affiliations:** a Educational Leadership & Policy Studies, Florida State University, Tallahassee, Florida, USA; b State Higher Education Executive Officers Association, Tallahassee, Florida, USA

**Keywords:** Advising, college courses, community colleges, developmental education, math

## Abstract

In 2014, developmental education became optional for many college students in Florida, regardless of prior academic preparation. This study investigated first-semester math course enrollment patterns for underprepared first-time-in-college (FTIC) students who would have previously been required to take developmental math and the passing rates for the students electing to take Intermediate Algebra (the most common gateway math course in Florida). We found that roughly a 3rd of underprepared students enrolled in developmental math, a 3rd enrolled in Intermediate Algebra, and roughly a 3rd enrolled in no math course whatsoever, with preparation level being related to enrollment pathways. Among those who enrolled in Intermediate Algebra, a small percentage also enrolled in developmental math in the same semester, either through a compressed or corequisite course, and FTIC students who received same-semester developmental support were more likely to pass Intermediate Algebra compared with similar underprepared students who took Intermediate Algebra without developmental support.

Far too many students graduate from high school underprepared for college-level work, and college readiness in math can often be the greatest obstacle to students’ success. Indeed, estimates have indicated that roughly 60% of community college students are referred to developmental math upon entry—a decision that could require a student to take a year or more of developmental courses before being able to enroll in credit-bearing, college-level classes (Bailey, Jeong, & Cho, 2010). Although developmental courses may provide necessary support to some underprepared students, a growing body of evidence suggests that students placed in developmental education, particularly those placed in developmental math, are highly unlikely to obtain an associate degree or transfer (Bailey et al., 2010; Calcagno, Crosta, Bailey, & Jenkins, 2007; Fong, Melguizo, & Prather, 2015; Melguizo, Hagedorn, & Cypers, 2008). In response, many states have begun to adopt policies that accelerate students into credit-bearing courses. Instead of requiring underprepared students to languish in multiple semesters of traditional developmental courses, some states have now either revised their placement policies to allow for greater flexibility in terms of who is required to take developmental courses or changed the way in which developmental courses are taught, often through accelerated course options (Bailey & Jaggars, 2016). In 2013, Florida did both by making developmental education optional for many students and changing the way developmental courses are taught for all students.

Since fall 2014, many community college students in Florida can enroll directly into introductory college-level (or *gateway*) courses regardless of prior academic preparation. In addition, all community colleges in the state must now offer developmental education courses through at least two of four approved instructional strategies, or modalities, including modalities that allow for enrolling in both developmental education and gateway courses in the same semester. In this study, we focused on math and used student-level data collected by the Florida Department of Education to investigate what happens when developmental education becomes optional and new instructional strategies designed to propel students through both developmental math and gateway math in the first semester of study are offered. Specifically, we asked: (a) What math courses do first-time-in-college (FTIC) students of varying academic preparation choose to take now that developmental education is optional? And (b) how successful are underprepared FTIC students who choose to take Intermediate Algebra in their first semester, while either skipping developmental education altogether or enrolling in a math course combination that allows for developmental education and Intermediate Algebra completion in the same semester?

We contribute to the existing literature in several unique ways. First, although significant research has been conducted on estimating student success at the cusp of placing into developmental education, by asking the “what if” question of what would happen if these students had bypassed developmental education, this study was able to capture enrollment pathways and associated success stories for FTIC students who would have previously been required to take developmental education but now have the option to skip it. Additionally, as some pathways allow students to take both developmental education and gateway courses in their first semester, we were able to examine whether students who would have been previously required to take developmental education might benefit from taking it and Intermediate Algebra instead of completely bypassing developmental education altogether. Finally, as developmental education policy reform is not unique to Florida, the findings from our work have important implications for policymakers and practitioners in other states as they contemplate changes to improve and streamline their developmental education programs.

## Florida context and recent reform

The 28 colleges in the Florida College System (FCS, formerly the community colleges) serve approximately 455,000 students (Florida Department of Education, 2016). Prior to the recent legislative reform, community college students in Florida, like those in most other states, were required to take a standardized placement exam upon entry. In Florida, the Postsecondary Educational Readiness Test (PERT) was the common placement tool for all students across the FCS and was used to place students either into college-level math, a one-course (upper-level) developmental math sequence, or a two-course (lower-level plus upper-level) developmental math sequence. More specifically, PERT scores ranged from 50 to 150 and students scoring 50 to 95 were placed into lower-level developmental math, students scoring 96 to 113 were placed into upper-level developmental math, and students scoring 114 or higher were placed in college-level math.

In 2013, the Florida legislature passed Senate Bill 1720, which made developmental education optional for many students while also changing how developmental education is taught for all students. Specifically, students who entered a Florida public high school in 2003 to 2004 or later and graduated with a standard high school diploma or any active-duty military personnel were given the option to bypass developmental education, regardless of academic preparation. In addition, these *exempt* students are no longer required to take a placement test. Nonexempt students (out-of-state students, graduates of private high schools, and those who entered ninth grade before 2003 to 2004, among others) are still required to take the PERT and follow the existing placement guidelines; however, the way in which developmental courses are taught also changed as part of the legislation. Specifically, FCS institutions were required to offer developmental courses in at least two approved instructional strategies: modularized, compressed, contextualized, and corequisite courses. As will be discussed in more detail in the following section, two of these options—compressed and corequisite courses—allow for a unique scenario not possible with the other modalities: completing both developmental education and gateway courses in the first semester of study. Many exempt students now have four options in their first semester: enroll in developmental education math, enroll in Intermediate Algebra, enroll in both developmental education and Intermediate Algebra, or enroll in no math course whatsoever.

Given that developmental education is now optional for exempt students, it is important to understand students’ enrollment choice patterns and how these choices are related to academic success. Previous studies of the Florida developmental education reform have revealed that overall, FTIC enrollment in developmental education courses declined following the policy change, enrollment in gateway courses soared, and while course-based passing rates in gateway courses declined, the net percentage of incoming students taking and passing gateway courses increased. That is, a higher percentage of all incoming students are passing gateway courses now that developmental education is optional. These trends were the most striking in math (Hu et al., 2016). These results suggest that some number of students who would have previously been placed in developmental education are taking and succeeding in gateway courses. In this study, we investigated this theory further by focusing specifically on the FTIC students who would have previously been placed into developmental education and by examining their enrollment patterns and the passing rates of students who elect to take Intermediate Algebra, . For those underprepared students who took Intermediate Algebra in their first semester, we also investigated whether there was any benefit from also taking a developmental education course in the same term, either through a compressed or corequisite format. We turn now to a further discussion of different delivery strategies that have recently been implemented in Florida and other states, including previous studies of their effectiveness.

## Course delivery modalities

A major component of the Florida legislation was offering developmental education courses that are contextualized and directly related to a student’s plan of study; developmental education courses that are shortened/compressed to fewer weeks than a standard term; developmental education courses that are taken concurrently with gateway courses; or developmental education courses that require students to progress only through specific topical areas, or modules, based on their individual needs. There is some evidence from other states on the effectiveness of these instructional strategies. In North Carolina and Virginia, for example, both states implemented multiple one-unit math modules in which students enroll based on their specific math needs (Kalamkarian, Raufman, & Edgecombe, 2015). Modular and contextualized courses still typically require that students enroll in at least one semester of developmental education coursework before enrolling in the gateway course. One feature of the Florida legislation is that it allows for options that enable students to enroll in both developmental education and gateway courses in the same semester, either through accelerated/compressed or concurrent/corequisite developmental education courses.

Accelerated—often called compressed—instruction typically limits time in developmental courses by shortening the length of the developmental education course and, in some cases, allowing students to progress into a likewise-compressed gateway course in the same semester. Acceleration has gained interest among practitioners, policymakers, and researchers because of the evidence that students are more likely to finish college if they more quickly make progress toward their degree (Bowen, Chingos, & McPherson, 2009). Accelerated programs have been associated with a higher likelihood of passing the course and enrolling in and passing the following course in the sequence (Guy, Cornick, Holt, & Russell, 2015; Hern, 2012; Jaggars, Hodara, Cho, & Xu, 2015; Jenkins, Speroni, Belfield, Jaggars, & Edgecombe, 2010; Sheldon & Durdella, 2009). Further, a cost–benefit analysis of an accelerated English program revealed that the accelerated program had a lower per-student cost than traditional remediation (Jenkins et al., 2010). However, one issue with the current understanding of the effects of accelerated remediation is that regression analyses cannot compare outcomes for students who chose to enroll in accelerated developmental education to outcomes for students who did not choose to enroll in the accelerated courses (Jaggars et al., 2015); our analysis, however, allows for such a comparison.

Corequisite developmental education, an instructional method touted by Complete College America (2016, p. 3) as “a proven bridge to college success,” provides students with developmental education courses while they concurrently enroll in the associated gateway course. Corequisite instruction is related to higher proportions of students passing college-level math courses across a variety of math achievement levels (Denley, 2015; Hayward & Willett, 2014), persistence to the next semester, and attempting and completing more college-level courses (Cho Kopko, Jenkins, & Jaggars, 2012). Colorado, Indiana, and Connecticut, among others, have recently implemented statewide policies to support or require corequisite instruction in their community colleges (Jones, 2015; Vandal, 2016). In this manuscript, we sought to understand not only what happens when developmental education becomes optional, but also whether there were benefits to enrolling in compressed or corequisite developmental education instead of bypassing developmental education completely for underprepared students who enrolled in Intermediate Algebra (gateway math) in their first semester.

## Research design

### Sample

Data for this analysis came from the Florida Education Data Warehouse (FL-EDW, a student-level state longitudinal administrative data set) and contain individual student records for all FTIC students who entered the FCS in fall 2014, the semester the reform went into effect. Because we were interested in enrollment patterns for students who had the option to bypass developmental education math, we excluded the nonexempt students from the analysis and included only those exempt students with complete transcripts from a Florida public high school. We could therefore include indicators for race, indicators for free/reduced-price lunch status, and separate indicators for whether a student took and passed high school Algebra 2, trigonometry, any other advanced math course, Honors English, and Advanced Placement (AP) English. Because we wanted to focus on underprepared students, we only included students who had a valid PERT score, a limitation we will discuss in a later section.

Our final analytic sample consisted of 20,591 students. Using the PERT scores, we then defined four groups: *severely underprepared* (PERT score 50–95; the students who would have been placed in the lower level of developmental math; 5,065 students), *moderately underprepared* (PERT score 96–106; the students who would have been placed in the upper level of developmental math but still far from the cut point; 6,308 students), *slightly underprepared* (PERT score 107–113; the students who would have been placed in the upper-level developmental education and close to the cut point; 3,930 students), and *college ready* (PERT score 114+; the students who would have been placed in college-level math; 5,228 students). Because some states give “bubble students,” or those close to the cutoff score, the opportunity to bypass developmental education (DE) and directly enroll in gateway courses, we divided the upper-level developmental education category (PERT score 96–113) into *moderately* (PERT score 96–106) and *slightly* (PERT score 107–113) underprepared. In doing so, roughly 40% of the upper-level DE students placed into the *slightly underprepared* category. Robustness checks using other cutoff scores ranging from 102 to 110 all yielded similar results in our full model.

### Math course choices

Using student course-taking records from the FL-EDW, we were able to determine whether FTIC students who would have previously been required to take developmental math prior to the reform took the following now that developmental education is optional: (1) no math course whatsoever, (2) developmental math, (3) Intermediate Algebra, or (4) both developmental education and Intermediate Algebra in the same semester. The FTIC students in Group 4 can be further disaggregated into two groups: (4a) students who took developmental education and Intermediate Algebra concurrently (corequisite developmental education) and (4b) students who took developmental education math as a separate course and then enrolled in Intermediate Algebra but did so in the same semester (via compressed courses). The main difference between these subgroups was that Group 4a had developmental education math and Intermediate Algebra *concurrently* and Group 4b had developmental education math and Intermediate Algebra as *discrete* courses. We coded enrollment in developmental education math for FTIC students enrolled in any nontransfer credit-bearing math course designated in the FL-EDW as developmental, and we coded enrollment in gateway math for students enrolled in MAT 1033: Intermediate Algebra, the most common statewide introductory credit-bearing math course in Florida.^1^ These two codes also allowed us to code Group 4, FTIC students enrolled in both developmental education and Intermediate Algebra. To delineate between Groups 4a and 4b, we made use of the primary delivery strategy indicators contained in the FL-EDW. FTIC students not enrolling in developmental education math or Intermediate Algebra were coded accordingly.

### Analytic strategy

To investigate math course enrollment patterns for underprepared FTIC students, we first made use of descriptive tables and figures, disaggregated by levels of preparation. For these analyses, we did not differentiate between Groups 4a and 4b. We also made use of a series of single-factor ordered logistic regression models that regressed the levels of preparation (severely, moderately, and slightly) on our measures of student background characteristics and high school course-taking indicators. We did so to explore whether there were differences in the composition of FTIC students identified as severely, moderately, and slightly underprepared; we present these findings as odds ratios with values greater than 1 associated with being classified into higher levels of preparation. Then, to inferentially examine the relationship between levels of preparation and enrollment patterns, we conducted a standard multinomial logistic regression specified as:

$$\mathrm{Pr}\left(Y_{i}=j\right)=\frac{exp\left(\beta_{j0}+\beta_{j1}moderate_{i}+\beta_{j2}slightly_{i}+\delta_{j}S_{i}+\gamma_{j4}HS_{i}\right)}{\sum_{k=0}^{K}exp\left(\beta_{j0}+\beta_{j1}moderate_{i}+\beta_{j2}slightly_{i}+\delta_{j}S_{i}+\gamma_{j4}HS_{i}\right)}$$

Under this specification, *Y_i_* is the math enrollment outcome *j* for individual *i, moderate* and *slightly* are dichotomous indicators (*severely* is the reference group), and *S* and *HS* are vectors of student demographic information and high school course-taking indicators. We present our results as relative risk ratios (rrr) and predicted probabilities of enrollment in the various pathways disaggregated by level of preparation (all other variables set to the within-group mean), with taking developmental education math as the base category. We also present the 95% confidence intervals for the predicted probabilities. Significant differences across levels of preparation can easily be identified if, for instance, the predicted probability for *slightly* underprepared falls outside the confidence interval for *severely* underprepared. The multinomial logistic regression model allowed us to estimate several equations simultaneously and to provide estimates of the relationship between levels of student preparation and math course-taking patterns. Put differently, this model allowed us to determine whether students in the *moderate* and *slightly* underprepared categories were any more or less likely to enroll in the four enrollment pathways compared to students in the *severely* underprepared category after accounting for student demographics and other measures of high school academic preparation.

Then, to determine the extent to which underprepared FTIC students were successful in the gateway course and how this relationship may have varied by enrollment option and level of preparation, we used a standard logistic regression equation. We included only FTIC students in Groups 3 and 4—those who enrolled in Intermediate Algebra—and compared their outcomes by enrollment pathway and by disaggregating Groups 4a and 4b in the following model:

$$Logit\left(Y_{i}\right)=\beta_{0}+\;\beta_{1}{\left(moderate\right)}_{i}+\;\beta_{2}{\left(slightly\right)}_{i}+\;\beta_{3}{\left(coreq\right)}_{i}+\;\beta_{4}{\left(compressed\right)}_{i}+\;\delta {\left(S\right)}_{i}+\;\gamma {\left(HS\right)}_{i}$$

Under this specification, *Y_i_* is a dichotomous indicator of whether student *i* passed Intermediate Algebra with a grade of C– or better and *moderate and slightly, S, and HS* are as before, while *coreq* and *compressed* are indicators for Groups 4a and 4b; the reference group is Group 3 (students who enrolled in gateway courses without any developmental education support). Our estimates for *moderate* and *slightly* are in comparison to *severely* underprepared, and our estimates for *coreq* and *compressed* are in comparison to no developmental education support. To make comparisons between *moderate* and *slightly* underprepared students as well as *coreq* and *compressed* pathways, we computed chi-squared statistics comparing the estimates against each other.

This model allowed us to examine whether FTIC students at different levels of preparation were successful in Intermediate Algebra and whether certain pathways may be more beneficial for underprepared students to pass Intermediate Algebra in the first semester.^2^ Put differently, this model allowed us to determine whether underprepared students who enrolled in Intermediate Algebra were any more successful if they also took developmental education math in the same semester, either in a discrete, compressed format or in a concurrent, corequisite format, compared to taking no developmental education course at all. In presenting these results, we again make use of predicted probabilities and associated 95% confidence intervals, disaggregated first by level of preparation and then by whether students took developmental education support in the same semester as when they took the gateway course.

### Limitations

As is always the case with analyses of existing observational data, our analysis was subject to limitations in terms of data availability and program structure. Most notably, as placement tests are now optional, there is some question over the generalizability of our findings, as students who may have previously been placed into developmental education based on their placement scores may not have taken the exam. To explore this, we conducted a series of binate *t* tests along the characteristics in the S (school) and HS (high school) vectors that compared 25,539 exempt FTIC students without a PERT score who were excluded from the analysis to the 20,591 FTIC students *with* a valid PERT score who were used in the analysis. Results from these analyses are provided in Table 1. Fewer FTIC students in our sample (those with PERT scores) had credit in Algebra 2, trigonometry, another advanced math course, Honors English, or AP English. Further, the study sample included significantly more Black students, female students, and students eligible for free/reduced-price lunch but fewer students who were Hispanic or of another race/ethnicity. Thus, FTIC students with PERT scores tended to have weaker high school records compared with exempt students without PERT scores, suggesting that our underprepared sample using PERT may be capturing a significant portion of underprepared FTIC students in the overall sample. Regardless, the FTIC students with valid test scores were an important subgroup to study as they are the students who, at some level, were likely aware that they would have previously been required to take developmental education math before taking Intermediate Algebra. In addition, previous research on the Florida developmental education reform has shown that advisors continue to rely on PERT scores as part of offering course enrollment guidance and encourage students without scores to take the PERT (Hu et al., 2015).

**Table 1.** Sample characteristics by level of preparation.

	(0)	(1)	(2)	(3)	(4)	(5)	(6)		
	Non-PERT Students	Study Sample	Severely Underprepared	Moderately Underprepared	Slightly Underprepared	College-Ready	Ordered Logistic Regression		
			(Math PERT 50–95)	(Math PERT 96–106)	(Math PERT 107–113)	(Math PERT ≥ 114)	OR	SE	Sig.
Student Background Characteristics (S)									
White	37.55%	38.10%	30.60%	38.20%	41.50%	42.50%	1.391	0.036	***
Black	20.73%***	23.00%	31.80%	23.00%	20.20%	16.50%	0.569	0.017	***
Hispanic	35.86%***	33.60%	33.80%	33.80%	32.90%	33.80%	0.994	0.026	
Other Race	5.85%*	5.30%	3.80%	4.90%	5.50%	7.10%	1.522	0.085	
Female	51.09%***	53.40%	57.50%	53.20%	54.40%	48.90%	0.818	0.021	***
Free/Reduced-Price Lunch	49.32%***	53.30%	57.60%	53.60%	51.80%	49.90%	0.817	0.021	***
High School Academic Preparation (HS)									
Algebra 2	83.34%***	74.40%	52.30%	70.00%	85.30%	92.90%	4.604	0.141	***
Trigonometry	6.01%***	3.90%	1.00%	2.50%	5.70%	7.00%	3.031	0.197	***
Other Advanced Math	25.05%***	14.70%	4.10%	8.90%	16.30%	30.60%	4.443	0.168	***
Honors English	58.7%***	46.70%	26.20%	43.90%	55.50%	63.30%	2.764	0.072	***
AP English	14.46%***	9.50%	3.20%	7.30%	11.80%	16.30%	2.802	0.122	***
*N*	25,539	20,591	5,065	6,308	3,930	5,288	20,591		

* p < .05. ** p < .01. *** p < .001.

*Note. *PERT = Postsecondary Educational Readiness Test; AP = Advanced Placement. Column 0 contains descriptive information on the 25,539 first-time-in-college (FTIC) students without a valid PERT score who were excluded from the study; the stars in Column 0 indicate the statistical significance from a series of bivariate *t* tests that compared these 25,539 FTIC students to the 20,591 FTIC students **with** a valid PERT score who were used in the analysis, along the characteristics in S and HS. Columns 2 to 5 disaggregate the study sample into the four levels of preparedness. Column 6 contains the odds ratios, standard errors, and significance levels from an ordered logistic regression model that regressed the four levels of preparation on the vectors S and HS.

Another limitation was related to both data availability and the structure of the reform. More specifically, one data point not collected by the state is the math course the student was advised to take. Though many do, students are not obligated to enroll in the courses suggested by their advisor. Previous research on the Florida developmental education reform has shown that not all students heed their advisors’ advice, a decision that is often influenced not purely by academic disagreement, but also by the location, instructor, and time of the course—all factors that have been shown to vary across the FCS (Hu et al., 2015). As such, our results are most appropriately interpreted as the enrollment patterns and FTIC student outcomes in an environment of increased choice—not a comprehensive analysis of student decision making.

## Results

### Levels of preparation

The descriptive statistics disaggregated by level of preparation (Table 1, Columns 2–5) and the odds ratios (OR) from the ordered logistic regression models (Table 1, Column 6) reveal several interesting findings. First, White and Black FTIC students were inversely and disproportionately represented across the preparation levels. White students comprised 30.6% of severely underprepared students and 42.5% of college-ready students, whereas Black students represented 31.8% of underprepared students yet only 16.5% of college-ready students (White OR = 1.391, *p *< .001; Black OR = 0.569, *p *< .001). Hispanic FTIC students, however, comprised nearly equal shares of the student population across the bands of preparation. The largest differences across the bands, however, were noticeable in terms of academic preparation. Nearly all (92.9%) college-ready students took Algebra 2 in high school compared with roughly half (52.3%) of severely underprepared students (OR = 4.604, *p *< .001). Similar differences, though somewhat smaller in magnitude, were observed across the other measures of high school academic preparation.

### Math enrollment patterns

Overall, only 34.9% of underprepared FTIC students took developmental education math, 27.7% took Intermediate Algebra, and 3.4% took both developmental education and Intermediate Algebra, while 34.2% took no math whatsoever. Table 2 disaggregates these patterns by level of preparation. Observationally, the findings in Table 2 suggest that the biggest differences in enrollment patterns across levels are in the share of FTIC students enrolling in developmental education math versus gateway math. Now that developmental education math is optional, 22.1% of slightly underprepared FTIC students enrolled solely in developmental education math compared with 45.2% of severely underprepared FTIC students. At the same time, 15.3% of the most severely underprepared FTIC students enrolled directly in Intermediate Algebra, while 43.0% of slightly underprepared FTIC students went directly into Intermediate Algebra. To further probe the relationship between preparation and enrollment choices, we turned to the multinomial logistic regression model and the predicted probabilities generated from it.

**Table 2.** Enrollment patterns by level of preparation.

	No Math		DE Math		Gateway Math		DE and Gateway Math		
	Number	Percent	Number	Percent	Number	Percent	Number	Percent	Total
Severely Underprepared	1,952	38.5%	2,289	45.2%	773	15.3%	51	1.0%	5,065
Moderately Underprepared	2,096	33.2%	2,180	34.6%	1,775	28.1%	277	4.4%	6,308
Slightly Underprepared	1,187	30.2%	868	22.1%	1,689	43.0%	186	4.7%	3,930
Total	5,235	34.2%	5,337	34.9%	4,237	27.7%	514	3.4%	15,303

*Note. *DE = developmental education. Slightly underprepared first-time-in-college (FTIC) students scored 50 to 95 on the Postsecondary Educational Readiness Test (PERT); moderately underprepared FTIC students scored 96 to 106 on the PERT; and slightly underprepared FTIC students scored 107 to 113 on the PERT.

Table 3 presents the rrr (the multinomial version of an OR) from our multinomial logistic regression analysis designed to determine how FTIC students’ preparation is related to math enrollment pathways; enrolling in developmental education was the reference group. Relative risk ratios greater than 1 indicate a positive relationship, while those less than 1 indicate a negative relationship. Across the board, the indicators for enrollment in Intermediate Algebra (rrr = 1.890, 3.776) or enrollment in both developmental education and Intermediate Algebra (rrr = 4.991, 7.625) were positive and statistically significant for moderately and slightly underprepared FTIC students, respectively. In other words, severely underprepared students were the most likely group to enroll in developmental education math. Interestingly, slightly underprepared students (our most prepared group) were more likely to enroll in no math at all than were the severely underprepared students compared with enrollment in developmental education math (rrr = 1.454).

**Table 3.** Multinomial logistic regression relative risk ratios.

	No Math	Gateway Math	DE and Gateway Math
Level of Preparedness			
Moderately Underprepared	1.067	1.890***	4.991***
	[0.048]	[0.103]	[0.786]
Slightly Underprepared	1.454***	3.776***	7.625***
	[0.083]	[0.235]	[1.278]
Student Background Characteristics (S)			
Black	0.975	0.835**	1.321
	[0.051]	[0.050]	[0.196]
Hispanic	0.958	0.993	2.632***
	[0.048]	[0.054]	[0.326]
Other Race	1.045	1.138	1.074
	[0.102]	[0.120]	[0.307]
Female	0.878**	0.902*	1.097
	[0.035]	[0.040]	[0.106]
Free/Reduced-Price Lunch	0.893**	0.947	1.097
	[0.038]	[0.044]	[0.113]
High School Academic Preparation (HS)			
Algebra 2	1.058	2.171***	2.332***
	[0.046]	[0.117]	[0.311]
Trigonometry	1.281	1.256	0.274**
	[0.171]	[0.165]	[0.127]
Other Advanced Math	1.210*	1.730***	1.305
	[0.100]	[0.135]	[0.199]
Honors English	1.126**	1.485***	1.460***
	[0.050]	[0.070]	[0.146]
Advanced Placement English	1.133	1.288**	0.849
	[0.102]	[0.113]	[0.164]
Constant	0.935	0.211***	0.006***
	[0.048]	[0.014]	[0.001]
chi2	1964.289		
*N*	15,303		

* p < .05. ** p < .01. *** p < .001.

Note. DE = developmental education. Included in this multinomial logistic regression model are the 15,303 first-time-in-college (FTIC) students in the sample identified as severely underprepared; severely underprepared is the reference group. Slightly underprepared FTIC students scored 50 to 95 on the Postsecondary Educational Readiness Test (PERT); moderately underprepared FTIC students scored 96 to 106 on the PERT; and slightly underprepared FTIC students scored 107 to 113 on the PERT. Estimates displayed are relative risk ratios; standard errors are in brackets.

With a few notable exceptions, student background characteristics appeared to be unrelated to math enrollment pathways. However, Black FTIC students were less likely than White FTIC students to enroll in Intermediate Algebra (rrr = 0.835), female FTIC students were less likely than male FTIC students to enroll in Intermediate Algebra (rrr = 0.902) and were less likely than male students to enroll in no math (rrr = 0.878) compared with enrollment in developmental education math. Also, low-income FTIC students were *less* likely to enroll in no math than were non-low-income students compared with enrollment in developmental education math (rrr = 0.893). In addition, FTIC students with stronger high school records were more likely to enroll in Intermediate Algebra instead of developmental education math, net of all other factors.

A more straightforward way to compare enrollment patterns by preparation across the different pathways was by examining predicted probabilities and their associated 95% confidence intervals. Table 4 provides predicted probabilities of enrolling in the different pathways, disaggregated by level of preparation. It is possible to identify statistically significant differences by identifying instances where confidence intervals do not overlap. For instance, slightly underprepared FTIC students were more likely to enroll in Intermediate Algebra overall as well as compared with severely and moderately underprepared FTIC students. Further, while severely underprepared FTIC students were the most likely to enroll in developmental education math, they were also more likely than moderately and slightly underprepared FTIC students to enroll in no math whatsoever. Finally, enrolling in both developmental education and Intermediate Algebra in the same semester was the least likely option across all ability levels, with severely underprepared FTIC students being the least likely of all to pursue this pathway.

**Table 4.** Predicted probabilities of enrollment pathways.

	No Math			DE Math			Gateway Math			DE and Gateway Math		
	Low	Est.	High	Low	Est.	High	Low	Est.	High	Low	Est.	High
Severely Underprepared	37.8%	39.2%	40.5%	44.3%	45.7%	47.1%	13.4%	14.3%	15.3%	0.6%	0.8%	1.1%
Moderately Underprepared	32.8%	34.0%	35.2%	33.8%	35.0%	36.2%	26.1%	27.3%	28.3%	3.3%	3.8%	4.3%
Slightly Underprepared	29.3%	30.7%	32.2%	20.7%	22.0%	23.3%	41.5%	43.1%	44.7%	3.5%	4.2%	4.8%

*Note. *DE = developmental education. Slightly underprepared first-time-in-college (FTIC) students scored 50 to 95 on the Postsecondary Educational Readiness Test (PERT); moderately underprepared FTIC students scored 96 to 106 on the PERT; and slightly underprepared FTIC students scored 107 to 113 on the PERT. The “Est.” column lists the estimated predicted probability while the “Low” and “High” columns list the lower bound and upper bound, respectively, of a 95% confidence interval around the estimated predicted probability.

### Success in gateway courses

Table 5 presents ORs from the logistic regression model predicting FTIC student success in Intermediate Algebra. In general, and perhaps unsurprisingly, better-prepared students were more successful in Intermediate Algebra. Both slightly underprepared (rrr = 3.174) and somewhat underprepared (rrr = 1.866) FTIC students were more successful than severely underprepared FTIC students, with slightly underprepared FTIC students being more successful than moderately underprepared FTIC students (chi2 = 62.68, *p *< .001). Presented as predicted probabilities, 23.4% of severely underprepared FTIC students passed Intermediate Algebra compared with 39.3% of moderately underprepared FTIC students and 54.3% of slightly underprepared FTIC students.

**Table 5.** Gateway completion for underprepared students.

Levels of Preparedness	
Moderately Underprepared	1.866***
	[0.179]
Slightly Underprepared	3.174***
	[0.310]
Enrollment Pathway	
Corequisite	1.380*
	[0.219]
Compressed	1.575***
	[0.189]
Student Background Characteristics (S)	
Black	0.898
	[0.080]
Hispanic	1.251**
	[0.096]
Other Race	0.972
	[0.143]
Female	1.384***
	[0.087]
Free/Reduced-Price Lunch	0.867*
	[0.058]
High School Academic Preparation (HS)	
Algebra 2	1.607***
	[0.148]
Trigonometry	2.589***
	[0.432]
Other Advanced Math	1.654***
	[0.146]
Honors English	0.966
	[0.064]
Advanced Placement English	1.246*
	[0.129]
Constant	0.170***
	[0.021]
chi2	426.766
*N*	4,731
*+ p *< .10. * p < .05. ** p < .01, *** p < .001.	

*Note. *Included in this logistic regression model are the 4,731 first-time-in-college (FTIC) students in the sample who took Intermediate Algebra and were identified as severely underprepared; severely underprepared is the reference group. Slightly underprepared FTIC students scored 50 to 95 on the Postsecondary Educational Readiness Test (PERT); moderately underprepared FTIC students scored 96 to 106 on the PERT; and slightly underprepared FTIC students scored 107– to 13 on the PERT. Estimates displayed are odds ratios; standard errors are in brackets.

In terms of student characteristics and measures of high school academic preparation, the results indicated that both are related to success in Intermediate Algebra. White and Hispanic students were more likely to pass Intermediate Algebra, as were female students, but those eligible for free/reduced-price lunch were less likely to pass the course. One point worth noting is that even students who were underprepared via their PERT scores benefited from taking rigorous math courses and AP English in high school. Students who took trigonometry, for example, were 2.6 times more likely to successfully complete Intermediate Algebra, even after controlling for their relative PERT math score.

When it came to same-semester developmental education support, underprepared FTIC students appeared to benefit from taking developmental education along with the gateway course instead of bypassing developmental education altogether, either through corequisite developmental education (rrr = 1.381) or compressed developmental education (rrr = 1.556). The difference in the estimates for corequisite and compressed developmental education, however, was not statistically significant (chi-squared = 0.47, *p *= .49). Although both are positive and are statistically significantly different from taking no developmental education support, there was no evidence to suggest whether either strategy is more beneficial than the other. Table 6 presents the associated predicted probabilities for passing Intermediate Algebra for underprepared FTIC students: 48.2% for students who took corequisite developmental education and 53.5% for students who took compressed developmental education along with Intermediate Algebra compared with 40.8% for underprepared students who took no developmental education support.

**Table 6.** Predicted probability of passing gateway math by gateway pathway.

	Pass Gateway Math		
	Low	Est.	High
Gateway Alone	39.2%	40.8%	42.3%
Gateway + Corequisite DE	40.7%	48.2%	55.8%
Gateway + Compressed DE	48.0%	53.5%	59.0%

DE = developmental education.

## Summary and discussion

Returning to our original research questions, we asked the following questions within the new Florida context where developmental education is now optional: (a) What math courses do FTIC students of varying academic preparation choose to take now that developmental education is optional? And (b) how successful are underprepared FTIC students who choose to take Intermediate Algebra in their first semester and either skip developmental education altogether or enroll in a math course combination that allows for developmental education and gateway course completion in the same semester? We attempted to answer these questions by employing student-level data and descriptive and multivariate regression analyses. Related to our first research question, we found that, indeed, severely underprepared FTIC students were the most likely group to enroll in developmental education math. Regarding our second research question, underprepared students appearred to benefit from taking developmental education along with Intermediate Algebra instead of bypassing developmental education altogether.

### Discussion

It is not surprising that many students who would have likely been placed into developmental math chose to skip it. In choosing developmental education they would be electing to take and pay for a class that does not earn them any credits, slows their progress, and has an uncertain impact on their future success. However, for some students, particularly the severely underprepared students, taking developmental education may be the best option. The fact that some of these severely underprepared FTIC students chose to skip developmental education is concerning. More concerning is that many underprepared FTIC students chose to skip math courses altogether. In fact, severely underprepared students had the highest probability of enrolling in no math whatsoever. This finding may have ramifications for future success when these students ultimately enroll in math courses and for their ultimate ability to graduate. For example, Johnson and Kuennen (2004) found that students who needed developmental math and took it prior to enrolling in an introductory course earned higher scores in the class and were less likely to fail the course. Further, additional research has shown that 48% of students who completed two college-level English courses and a college-level math course within their 1st year completed a credential within 6 years, compared with just 18% of students who did not meet these gateway milestones within the 1st year (Denley, 2016). Similarly, Nora, Barlow, and Crisp (2005) found that more students who enrolled in a gateway math course within their 1st year graduated within 6 years when compared with students who failed or withdrew.

Our results seem to provide some validation of the PERT cutoff scores but also demonstrate that high school academic records are predictive of student success beyond what the PERT score is able to capture. That is, we found that FTIC students who had higher PERT scores and were only slightly or moderately underprepared tended to do better in Intermediate Algebra compared with students who were significantly underprepared. Further, our results demonstrate that some magnitude of students who would have been previously required to take developmental education based solely on their PERT score were successful in passing gateway math. This finding offers support to numerous other studies that have shown the benefit of using multiple measures (e.g., Ngo & Kwon, 2015; Scott-Clayton, Crosta, & Belfield, 2014). A combination of flexible cutoff scores (or bands) with multiple measures such as high school transcript information (course taking and grades) might be the best approach to developmental education placement. Future research ought to explore how to construct a multiple-measures policy that best positions students for success.

A number of policy and advocacy organizations have advocated for developmental education instructional approaches that allow students to earn college-level credit while receiving supplemental developmental education support simultaneously or in a compressed format in the same semester as a likewise-compressed gateway course. We found support for both corequisite and compressed developmental education. Our results suggest that FTIC students who took advantage of these approaches have a higher likelihood of passing Intermediate Algebra than those who went directly into pure college-level math courses without supplemental developmental education support. However, the utilization of these approaches is very low in Florida. Future research will need to examine why this is the case. Are students intimidated by the prospect of trying to do both in one semester? Are not enough colleges offering the courses in ways that are accessible to students? Is it a combination of the two or something else?

Along these lines, our findings also raise equity questions. We are left with questions as to why Black students and female students were less likely to enroll in gateway math, particularly because we controlled for a number of other student characteristics and prior academic preparation. Might academic advising differ for traditionally underrepresented students? And how might these disparities be related to future outcomes such as majoring in science, technology, engineering, and mathematics fields, where Black students and female students are also historically underrepresented?

## Policy considerations and next steps

Considering these issues and the findings from our study, we offer the following considerations for policy, practice, and next steps. First, our results highlight the potential importance of advising in the new context of increased student choice. When students are afforded significant discretion over their course enrollment options, colleges and states ought to invest in advising centers and practices. Having enough advisors, providing the advisors with proper training, and ensuring that they have enough resources may be critical to helping students make the best choices possible in their first semester. Our results indicate that even in the first semester, the pathways students choose—whether into developmental education, whether to enroll in college-level courses with or without supplemental developmental education support, or whether to skip math all together—are related to their likelihood of passing college-level math in the first semester.

Second, the placement exam, although not perfect, appears to provide valuable information, which when combined with students’ relevant high school transcript information, may improve student placement. Colleges would do well to continue to offer the exam and to advise students to take it. Given that the exam is now optional in Florida, colleges may also want to remove potential barriers to taking the exam such as waiving the cost of the exam, providing incentives, and providing easy access to the exam (such as allowing immediate test taking in the advising center) to encourage more students to take the exam.

Third, college staff need to be aware that some students are delaying taking math in the first semester. Although colleges cannot mandate that students take developmental education, perhaps they should consider mandating that students take a math course in their first semester, with developmental math as one option to fulfill that requirement. Delaying math may be related to more limited success, and mandating that students take it early in their college career may help more students successfully pass math and move toward graduation.

Fourth, colleges may want to expand the delivery of corequisite and compressed developmental education and encourage more students to enroll in these options. Corequisite and compressed instructional techniques are associated with increased student success in gateway courses; however, in Florida, they appear underutilized. In Florida, and potentially in other states, state officials ought to provide greater clarity regarding what constitutes corequisite and compressed developmental education and should provide additional technical assistance regarding how best to design and deliver these forms of developmental education. We note, however, that although we have controlled for a number of student-level characteristics, there may be factors (such as motivation) that may be related to students selecting these modalities over others. Thus, future research, ideally using randomization, is vital to more fully understand the causal impacts of corequisite and compressed instructional strategies.

Fifth, future research is warranted on the experiences of traditionally underrepresented students following the recent reform. Studying implications of the reform on equity is essential for a complete understanding of student outcomes. Specifically, future research should address questions of whether academic and career advising differs for students based on their race/ethnicity or gender, their level of math preparation, and their early math success.

Sixth, future research is desperately needed on outcomes beyond the first semester, including research that makes use of quasiexperimental or experimental designs. For example, is taking compressed or corequisite developmental education a “better” option than enrolling in a whole semester of developmental education and trying to take Intermediate Algebra in the spring? Examining gateway success within the 1st year may provide additional insight into student success, with or without developmental coursework. It provides students the flexibility to enroll in successive developmental and gateway courses (one each semester) and still complete the gateway requirement within the 1st year. It also allows for students to delay math enrollment by just one semester.

## Conclusion

The Florida developmental education redesign provides underprepared FTIC students with a variety of options not available in most other contexts, thereby setting the stage for policymakers, practitioners, and researchers to work together to identify ways to improve student success, both in Florida and beyond. By utilizing data from the state administrative longitudinal data system, this study sheds important light on what happens when developmental education is optional for underprepared students. Although not all underprepared FTIC students were successful in Intermediate Algebra, some were, particularly those who were only slightly underprepared and those who took same-semester developmental coursework along with the gateway course. These results present a cautiously optimistic view of optional developmental education that we will continue to study. Underprepared FTIC students can be successful in Intermediate Algebra; however, future research is needed on how to best ensure their success.

## Funding

The research reported here was supported by the Institute of Education Sciences, U.S. Department of Education, through Grant R305A160166 to Florida State University and in part by a grant from the Bill & Melinda Gates Foundation. The opinions expressed are those of the authors and do not represent views of the institute, the U.S. Department of Education, or the Gates Foundation.

## Notes

1. The FCS has common course numbering, and the traditional and most common gateway mathematics course is Intermediate Algebra (MAT 1033). There are, however, additional mathematics and statistics courses that can fulfill the gateway requirement, depending on a student’s intended major. Investigating outcomes in these courses will be covered in a future analysis.

2. In an additional iteration of the model, we interacted the levels of preparation with the course delivery methods to determine whether there were any differential relationships of the delivery methods on gateway success for students of varying ability. None of the interactions were statistically significant, and a likelihood ratio test showed that the added interactions did not significantly improve model fit. For these reasons, we do not present these results.
